# Disparities in Patient Demographics at a Student-Run Free Clinic: Comparing Clinic Utilization to City, State, and National Trends

**DOI:** 10.1007/s10900-024-01437-3

**Published:** 2025-01-20

**Authors:** Katherine Esser, Johnny McKeown, Tatiana White, Steuart Besly, Julianna Sim, Addison Sparks, Sydney Hatch, Richard Paat, Coral Matus

**Affiliations:** Department of Medicine, University of Toledo College of Medicine and Life Sciences, 3000 Arlington Ave, Toledo, OH 43614 USA

**Keywords:** Free clinic, Student run free clinic, Racial disparities, Socioeconomic status, Uninsured

## Abstract

**Background:**

With 8.4% of Americans uninsured, free clinics serve as essential safety nets for underserved populations. This study compared the demographics of the patients of a student-run free to Toledo, Ohio, and national census data to evaluate health needs, barriers to care, and the characteristics of the underserved population.

**Methods:**

A retrospective review of 1,338 visits across five clinic sites was conducted from February 2023 to February 2024. Demographic variables, including race, insurance, education, sex, and primary language, were analyzed and compared to 2020 Census data from Toledo, Ohio, and the United States.

**Results:**

Compared to Toledo, the clinic served 22.63% fewer White patients, 17.27% more Hispanic/Latino patients, and 5.62% fewer African American patients. Among clinic patients under 65 years, 61.91% were uninsured, compared to 8.3% in Toledo and 10.2% nationally. Non-English speakers were overrepresented (33.4% vs. 6.8% in Toledo and 9.5% nationally), with Spanish speakers making up 21.72% of clinic patients compared to 3.2% in Toledo. Educational attainment differed slightly, with fewer high school graduates (82.97% vs. 87.10% in Toledo) but more individuals with higher education degrees (27.10% vs. 19.6% in Toledo and 25.6% nationally).

**Conclusions:**

This study underscores the demographic differences between CCC patients and city, state, and national populations, offering insight into the populations most reliant on free clinics. Policymakers and public health agencies must consider these disparities to tailor interventions addressing healthcare access and social determinants of health.

## Introduction

The United States comprises approximately 1,400 free clinics that served over 5.8 million patients collectively in 2022 [1]. With reports between 2022 and 2023 showing that 27.6 million (8.4%) of Americans of all ages did not have health insurance, these free clinics often serve as public safety nets [2]. The demographics of patients that receive care at these clinics has been reported [3–8]. However, there is a paucity of data regarding how these patients compare to their communities at large.

A previous study at a free clinic described over 78% of their patient population as uninsured and cost being a primary barrier to receiving care from a traditional healthcare facility [9]. The Affordable Care Act (ACA) of 2010 aimed to increase healthcare access for the 47 million Americans who were uninsured at the time [10]. Medicaid was expanded to adults who earned up to 138% of the federal poverty level [10]. In 2012, the United States Supreme Court made this Medicaid expansion optional for states [10]. As of 2024, 40 states expanded their Medicaid program, including Ohio [11]. Ohio’s Medicaid expansion took effect January 1, 2014, and has since allowed more than 1.26 million patients access to healthcare [12]. However, more than 609,000 patients then lost coverage in 2023 during the re-enrollment period following the end of the COVID-19 public health emergency [13]. With the various attempts to repeal the ACA, it is possible that millions more patients nationally might find themselves without insurance, and free clinics could serve as a safety net for these patients [14].

Free clinics have made efforts to achieve health equity by closing the gaps in health care disparities, specifically among disadvantaged populations. The CommunityCare Clinics (CCC) is a student-run free clinic operating five sites in Toledo, Ohio. The CCC provides over 4,000 clinic visits each year with a team of 300 active volunteers that include providers and students in various health professions programs affiliated with The University of Toledo. Each clinic site has served as a de-facto primary care office for the majority of its patients where all services offered are free of charge.

As CCC and similar free clinics continue to expand, it is necessary for its services to reflect the current social determinants of health and health disparities the patient population encounters. Responses retrieved from patient intake forms were compared to the most recent 2020 census report based out of Toledo, OH. This study aims to determine the demographics of our community to characterize the health needs of the area, barriers to care, and characteristics of the underserved population.

## Methods

### Survey Administration

Patients who completed an intake form at our five free clinics from February 2023 to February 2024 were included. All patients completed an intake form prior to being seen for care. Demographic parameters extracted from reported information on the intake form were race, age, sex, education level, health insurance status, and primary language spoken.

### Statistical Analysis

The collected information was compared to the available 2020 Census information from the city of Toledo, the state of Ohio, and national data of the United States. Quantitative variables were evaluated using a chi-squared test. *P*-values < 0.05 were considered statistically significant. Significant values were then sub-analyzed by Z-test for two proportions comparing the CCC’s results to that of the city, state, and nation. All statistical analysis was completed in SPSS software (Version 29, IBM Corp., Armonk, NY). Due to the survey nature of the data collection, there were some missing data values that were excluded from the statistical analysis. Incomplete surveys were included in analysis for the questions answered. This study was approved by the Institutional Review Board (301994-UT) at the University of Toledo and adhered to its guidelines.

## Results

### Clinic Demographics

A total of 1,338 visits met the inclusion criteria, including 737 unique visits, and 601 follow up visits. The male-to-female proportion of patients served at the clinic were almost equal, at 49.93% and 50.07%, respectively. In regards to race, the majority of patients were White, comprising 33.37% of total patients seen. This was followed by Hispanic or Latino, comprising 26.07% of patients seen, and then African American, comprising 21.48% of patients. The age composition of the clinic was 1.02% under 5, 4.25% under 18, 77.70% age 18–65, and 20.20% 65 and over. The primary language for 66.60% of patients seen was English, followed by Spanish at 21.72%. The majority of patients achieved a high school diploma or higher, at 82.97% of patients. 70.21% of patients under 65 stated they did not have health insurance. Of the 29.79% of the clinic patients that were insured, the primary reasons for attending a free clinic included inability to afford copays, 33.89%, and inability to wait for a primary care appointment, 27.69%.

### Clinic Versus City, State, and National Demographic Comparisons

With the exception of sex, all areas analyzed had a statistically significant difference in the population being served at the free clinic compared to the population of the surrounding city of Toledo, Ohio [15, 16]. Comparison demographics are summarized in Table 1.

**Fig. 1:**
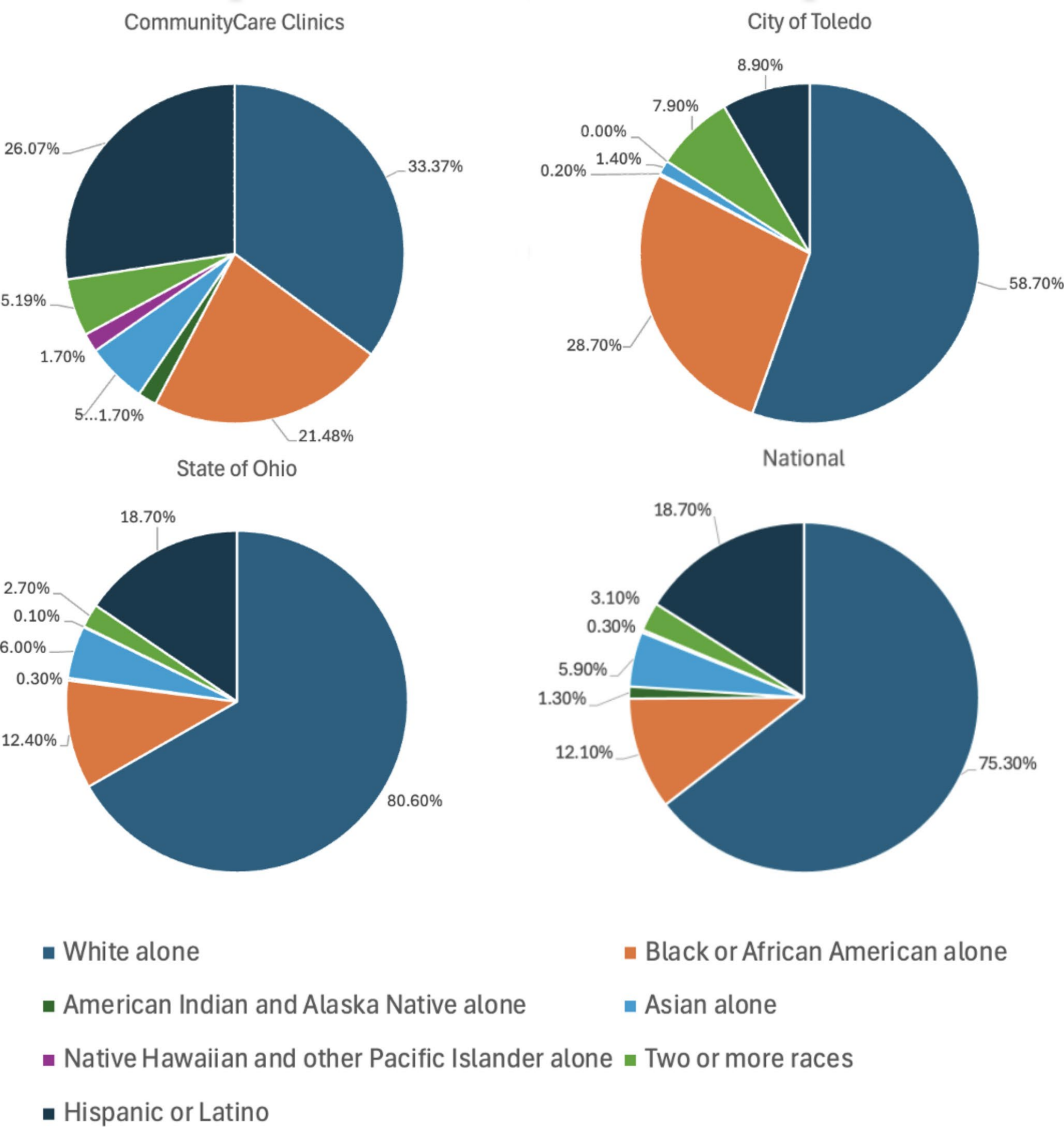
Pie-chart comparisons of race between the patients of the free clinic and city, state, and national demographics

The largest differences seen between the clinic’s patient profile compared to the surrounding city’s profile in race were in the Hispanic or Latino populations and White populations. Compared to the surrounding city population of 8.8% consisting of Hispanic or Latino, or state and national proportions of 18.7%, 26.1% of the clinic’s population consisted of Hispanic or Latino. Additionally, the White population of Toledo is 58.7%, versus the 33.4% of the clinic’s population (Fig. 1). The clinic served a patient population that was 21.48% black, which was lower than the proportion in the city at 28.7%, but more than double of the state and national proportion at about 12.0%. Compared to the average rate of insured persons being about 91.0% at the city, state, and national level, only 29.8% of the clinic patient’s under the age of 65 indicated that they had health insurance. Of the 29.8% of the clinic patients that were insured, the primary reasons for attending a free clinic included inability to afford copays, 33.9%, and inability to wait for a primary care appointment, 27.7%. The clinic served the highest portion of patients between the ages of 18–65 at 77.70% compared to the proportion of this age group at the city, state, and national level being about 54.0%. The clinic also had a lower portion under age 18 at 4.4% compared to the city, state, and national levels of around 20.0%. Finally, there was a large difference in the primary language spoken for those seen at the free clinic compared to the surrounding city. The largest differences being only 66.6% reported their primary language as English compared to approximately 87.0% proportion reported at the city, state, and national levels. Additionally, 21.7% of the clinic population reported their primary language of Spanish compared to around 3.0% for the city and state, and 13.7% at the national level.

**Table 1:** Comparison of demographics between the patients of the free clinic and the surrounding city, state, and nation

	Community Care Clinics	Toledo Census	*p-value	Ohio Census	*p-value	National Census	*p-value	Chi-Square *p*-value
**n**	1338	270,880		11,799,331		331,464,948		
**Sex**								
Male	668 (49.93%)	132,460	0.67448	5,817,070	0.56868	164,075,149	0.67448	
Female	670 (50.07%)	138,420	0.45326	5,982,261	0.64552	167,389,799	0.75656	**< 0.001**
**Race**								
White alone	446 (33.37%)	159,007	**< 0.00001**	9,510,261	**< 0.00001**	249,593,106	**< 0.00001**	
Black or African American alone	287 (21.48%)	77,743	**< 0.00001**	1,463,117	**< 0.00001**	40,107,259	**< 0.00001**	
American Indian and Alaska Native alone	23(1.70%)	542	**< 0.00001**	35,398	**< 0.00001**	4,309,044	0.17702	
Asian alone	73(5.49%)	3,792	**< 0.00001**	707,960	**0.4009**	19,556,432	0.4902	
Native Hawaiian and other Pacific Islander alone	23(1.7%)	0	**< 0.00001**	11,799	**< 0.00001**	994,395	**< 0.00001**	
Two or more races	69(5.19%)	21,400	**0.0002**	31,8582	**< 0.00001**	10,275,413	**< 0.00001**	
Hispanic or Latino	349(26.07%)	241,08	**< 0.00001**	2,206,475	**< 0.00001**	61,983,945	**< 0.00001**	**< 0.001**
**Age**								
Under 5	14 (1.02%)	17,607	**< 0.00001**	660,763	**< 0.00001**	18,230,572	**< 0.00001**	
Under 18	58 (4.35%)	62,302	**< 0.00001**	2,584,053	**< 0.00001**	71,927,894	**< 0.00001**	
18–65	1040 (77.7%)	148,984	**< 0.00001**	6,348,040	**< 0.00001**	182,637,186	**< 0.00001**	
65 and over	270 (20.2%)	41,986	**< 0.00001**	2,206,475	0.16452	58,669,296	**0.01732**	**< 0.001**
**Health Insurance Status**								
Without Health Insurance under 65	939	22,483	**< 0.00001**	873,150	**< 0.001**	31,489,170	**< 0.001**	
With Health Insurance under 65	399	248,397	**< 0.00001**	10,926,181	**< 0.00001**	299,975,778	**< 0.00001**	**< 0.001**
**Education Level**								
High School graduate or higher	1110	235,936	**< 0.00001**	10,808,187	**< 0.00001**	296,329,664	**< 0.00001**	
Less than High School graduate	228	34,944	**< 0.00001**	991,144	**< 0.00001**	35,135,284	**< 0.00001**	
Bachelor’s degree or higher	363	53,092	**< 0.00001**	3,645,993	**0.00288**	116,012,732	**< 0.00001**	
Less than bachelor’s degree	975	217,788	**< 0.00001**	8,153,338	**0.00288**	215,452,216	**< 0.00001**	**< 0.001**
**Language**								
English	891	252,460	**< 0.00001**	10,831,786	**< 0.00001**	275,115,907	**< 0.00001**	
Spanish	291	8,668	**< 0.00001**	330,381	**< 0.00001**	45,410,698	**< 0.00001**	
Indo-European	54	3,251	**< 0.00001**	342,181	**0.01314**	12,927,133	**0.01314**	
Asian/Islander	47	2,167	**< 0.00001**	165,191	**< 0.00001**	11,932,738	**< 0.00001**	
Other	56	4,063	**< 0.00001**	141,592	**< 0.00001**	4,309,044	**< 0.00001**	**< 0.001**

* *p*-values calculated from Z-test for two proportions

## Discussion

This study identified significant differences in the demographics of patients at our free clinic compared to the surrounding city, state, and national populations, with the exception of sex. While many studies have investigated the demographics of free clinic patients, few have compared these directly to the demographics of the cities they serve [3–5, 7–9, 17–19].

A surprising finding was the lower proportion of Black patients served at our clinic compared to the city of Toledo (21.48% vs. 28.1%). This finding contrasts with prior studies showing wide variability in the proportion of Black patients served by free clinics, ranging from 14 to 87% [3–5, 7, 9, 18]. Interestingly, this variation does not appear to correlate directly with city demographics. For example, Cadzow et al. reported that a free clinic in Buffalo, NY, served 87% Black patients, despite only 33.2% of the city’s population being Black, while a clinic in Portland, OR, served 50.6% Black patients despite the city’s Black population being only 5.9% [4, 15, 19].

Although our clinic serves a higher proportion of Black patients than both state and national averages (12.40% and 12.10%, respectively), the disparity compared to Toledo’s Black population highlights areas for improvement. Several factors may explain this finding. One potential contributor is disparities in cultural concordance between the patient population and clinic volunteers. Evidence suggests that patients are more likely to seek care and feel comfortable with providers who share their racial or ethnic background [20–22]. However, like many free clinics, our clinic has a limited number of Black physicians and student volunteers, reflecting the broader underrepresentation of Black individuals in medicine. According to recent statistics, Black physicians make up only 5.7% of the U.S. physician workforce, and Black students account for 9.2% of medical school matriculants [23]. These disparities present a significant challenge in achieving diverse representation among clinic staff.

Another consideration is that socioeconomic factors, while critical, do not entirely explain health-seeking behaviors. Black and Hispanic Toledoans experience poverty at similar rates, 31.4% and 34.1%, respectively, compared to 15.5% among White residents [15]. Despite these similar poverty rates, we serve a disproportionately high number of Hispanic patients compared to their representation in the city, while the proportion of Black patients served remains lower than expected. This suggests that additional social determinants of health, such as historical mistrust of healthcare institutions, cultural stigmas surrounding certain types of care, and the legacy of structural racism, may influence engagement with our clinic.

Efforts to address these barriers, such as a new partnership with the Student National Medical Association (SNMA), aim to increase Black representation among clinic volunteers and build trust within Black communities. Future initiatives will include community listening sessions, faith-based outreach, and partnerships with advocacy organizations to address barriers to care. Ultimately, these findings highlight the need for free clinics to examine not only structural factors, such as volunteer demographics, but also the broader social determinants that influence health-seeking behaviors among Black patients. Our experience underscores the importance of aligning clinic operations with the unique needs of the populations they aim to serve, particularly in the context of historically underserved communities. Further research is needed to explore whether targeted efforts to increase cultural concordance and community engagement can lead to sustained improvements in clinic utilization by Black patients.

We served proportionately 2.8 times more Hispanic patients than would be expected based on the demographics of the city. There was also variation in the literature of the proportion of Hispanic patients served in free clinics across the country, ranging from 3 to 70.1% [3–5, 7, 9, 18]. This finding could be due to income disparity. Despite the fact that Hispanics are employed at the same rate as African Americans and Whites, they make 55% of what Whites do, and 68% of what African Americans do [24]. Hispanics are also less likely to be employed at jobs that provide employer-sponsor insurance- a 2018 study found that 66% of whites, 46% of Blacks, and 41% of Latinx are covered by employer-sponsored health insurance [25]. The disproportionately higher population of Hispanics served in comparison to the city of Toledo may be attributed to our partnership with a local church. This church is the only one in the city that conducts Mass in Spanish, and we decided to host a clinic here specifically to serve the Hispanic population. This data shows our outreach to the Hispanic community has been successful. We also observed that 22.51% of patients at our clinic primarily spoke Spanish, 7.1 times higher than the proportion of Spanish-speaking residents in Toledo. This emphasizes the need for consistent, qualified medical interpreters. Evidence shows that language barriers negatively impact patient satisfaction, understanding of medical instructions, and adherence to care plans, while also increasing clinicians’ likelihood of misdiagnosis or excessive testing [26–29]. Ensuring access to medical interpreters could improve care quality and patient retention.

Another notable finding was the lower proportion of White patients served (34.37%) compared to Toledo’s White population (56%). This may reflect socioeconomic factors, as White residents in Toledo are more likely to have employer-sponsored insurance [25, 30]. However, it also highlights that insurance coverage does not guarantee access to care. Nearly one-quarter (24.72%) of patients at our clinic were insured but cited financial barriers, such as copays and deductibles, as the primary reason for seeking free care. Despite federal efforts to increase insurance coverage, including the Affordable Care Act, significant gaps in access to care remain, particularly for low-income populations [31, 32]. The presence of insured patients at free clinics underscores the limitations of current insurance models and the need for additional safety nets for vulnerable populations.

Publishing demographic data such as this study’s findings is crucial for directing public health resources and identifying national trends. Understanding the disparities between free clinic patient populations and broader community demographics allows clinics to better align their services with community needs. Addressing issues such as cultural concordance, targeted outreach, and access to interpreters is essential for improving care for underserved populations. Future research should explore whether these strategies lead to sustained improvements in clinic utilization and health outcomes.

## Conclusion

This study is unique in its attempt to analyze the concordance and discordance of patient demographics with the demographics of the city it serves. This free clinic served a disproportionately high number of Hispanic patients, and a disproportionately low number of Black and White patients. A significant proportion of patients listed their first language as Spanish. A disproportionate amount of patients were uninsured, with those who were insured citing inability to afford copays or inability to wait for a primary care appointment as the reason they sought care at the free clinic.

This demographic research may suggest there is still work to be done for the free clinic to better serve the African American patient population. It reinforces the need for care in Hispanic and Spanish-speaking populations, and validates outreach in these communities as well as increased initiatives providing Spanish interpretations at clinics. This information can be used by public health agencies to appropriately direct resources and increase health education with the goal of increasing access to medical care for the underserved.
